# Apparent trends in the use of femoral megaprostheses: an analysis from the National Joint Registry

**DOI:** 10.1186/s42836-022-00150-7

**Published:** 2022-12-01

**Authors:** Darren R. Puttock, Daniel P. Howard, Nicholas C. Eastley, Robert U. Ashford

**Affiliations:** Leicester Orthopaedics, University Hospitals of Leicester, Leicester, UK

**Keywords:** Femoral Megaprosthesis, Trends, Indication, Registry data

## Abstract

**Background:**

Megaprosthetic replacement (MPR) of the femur is typically reserved for salvage or oncological reconstruction. Presently little is known about the provision of femoral MPRs performed nationally, the trends in indications for their use, and their outcomes beyond published unit-level data. Although the National Joint Registry (NJR) collects data as part of a mandatory arthroplasty audit process, MPR data entry on this platform is thought to be inconsistent. The aim of this study is to determine current trends for femoral MPR procedures as submitted to the NJR.

**Methods:**

Data for all procedures submitted to the NJR using the following implants were extracted: METS (Stanmore/Stryker), MUTARS (Implantcast), Segmental (Zimmer), GMRS (Stryker) and MEGA C (LINK). Pseudoanonymized data were analyzed through the NJR’s research Data Access Portal and are reported using descriptive statistics.

**Results:**

A total of 1781 procedures were identified. Submitted cases increased for primary and revision hip and knee categories over the study period, although they plateaued in recent years. MPR implants were most commonly used in revision hip arthroplasty procedures. MPR use for the management of peri-prosthetic fractures has increased and now represents the most commonly reported indication for MPR use in both hip and knee revision categories. Few centers submitted large MPR case volumes (which were noted to be lower than published unit case series, indicating NJR under-reporting), and the vast majority of centers submitting MPR cases did so in low volume.

**Conclusions:**

Due to the limitations identified, reported case volumes must be interpreted with caution. An MPR-specific NJR data entry form has been developed to allow more accurate and tailored reporting of MPR procedures, to support specialist service provision, and to provide meaningful data for future research.

**Supplementary Information:**

The online version contains supplementary material available at 10.1186/s42836-022-00150-7.

## Introduction

Megaprosthetic (mega-endoprosthetic) replacement (MPR) of the femur using modular megaprostheses has long been an established method of treating orthopedic malignancies [1], with the first implant designed for this purpose described over 70 years ago [2]. Advances in MPR design, technologies and surgical techniques mean that megaprostheses now form the mainstay of skeletal reconstructions following limb salvage surgery for primary malignant tumors in the appendicular skeleton [3]. Due to their ability to overcome severely diminished bone stock, femoral MPRs also play an established role in the management of select complex revision arthroplasty and trauma cases [3, 4].

**Fig. 1:**
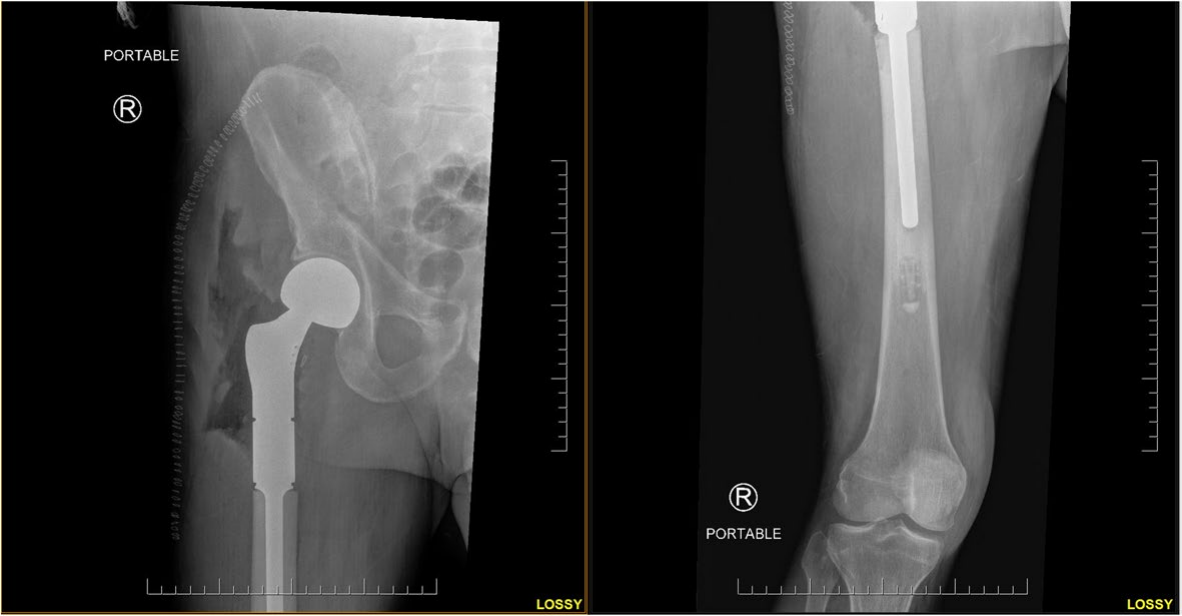
An AP radiograph showing a proximal femoral replacement using a megaprosthesis implant

An endoprosthesis is an artificial device, which is placed inside the body to replace a missing body part [5]. A megaprosthesis is a modular endoprosthetic implant used to replace large sections of bone extending into metaphyseal and diaphyseal sections, which require removal due to tumor reconstruction or other pathologies, with poor bone stock (Fig. 1).

The increasing popularity of femoral megaprostheses is a result of numerous advantageous characteristics, such as allowing early weight-bearing, rapid restoration of function and improved cosmetic appearances when compared to alternative treatment methods [6]. In cases of fracture or reconstruction following malignancy, MPRs avoid reliance on union of pathological bone [7]. Disadvantages of femoral MPR include their failure rates and high initial costs. However, these costs remain low for oncological reconstruction when compared to the ongoing care cost of limb amputation, which traditionally was the only option for this patient cohort [8].

Within the healthcare systems of England, Wales, Northern Ireland and the Isle of Man, it is mandatory to submit arthroplasty procedure data to the National Joint Registry (NJR) [9, 10]. Due to difficulties recording megaprosthesis data within the NJR’s existing structure and because of concerns regarding the publication of outcome data in the context of malignancy and salvage surgery, MPR data entry on this registry is thought to be inconsistent.

The primary aim of this study was to report current trends in femoral megaprosthesis use, using data submitted to the NJR. An analysis of centers contributing to this dataset was also undertaken to gain further insight into MPR case volumes and specialist care provision. Using published case series and unit-level data, we also sought to ascertain whether this NJR data are likely to be accurate.

## Methods

An application was made to the NJR and Healthcare Quality Improvement Partnership (HQIP) for all data involving a femoral megaprosthesis submitted between the 1st April 2003 and the 1st September 2020. Pseudoanonymized data were extracted for hip primary, hip revision, knee primary and knee revision procedures for the following implants: METS (Stanmore/Stryker), MUTARS (Implantcast, Birmingham, UK), Segmental (Zimmer,Warsaw, Indiana), GMRS (Stryker, Kalamazoo, Michigan) and MEGA C (LINK, Hamburg, Germany). Datasets included procedure indication, date of surgery and pseudonymized operating center. Analyses were performed through the NJR's secure Data Access Portal using descriptive statistics to determine temporal trends of case volumes, indications, and submitted case volume by the surgical center.

Due to anticipated data paucity and bias, outcomes (survival, revision, or death) were not analyzed.

## Results

A total of 1781 procedures were identified and included (involving 632 males and 1149 females), from patients with a mean age of 74.48 years (standard deviation = 12.36). A further breakdown of patient demographics in each procedure category is provided in Table 1.

**Table 1:** Patient demographics by procedure type

Procedure type	Male:Female	Mean Age (years) (SD)
Hip Primary	152:254	69.72 (14.01)
Hip Revision	245:413	75.58 (11.71)
Knee Primary	56:153	74.33 (12.83)
Knee Revision	179:329	76.97 (10.39)

**Fig. 2:**
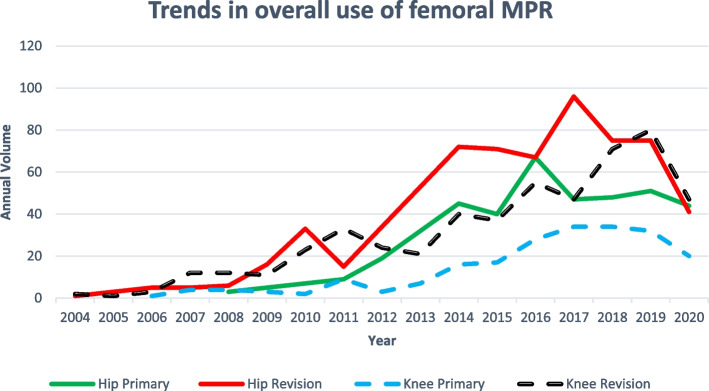
Trends in megaprosthesis use by procedure type

The overall number of NJR case entries relating to femoral megaprostheses increased over the study period, excluding 2020. The decrease in submitted case volume in 2020 remained true when accounting for the fact that data were available for the first eight months of this calendar year only. This general increase was seen across all procedure types (Fig. 2).

Megaprosthesis entries were most common for revision hip procedures, peaking at 96 cases in 2017, closely followed by revision knee procedures which peaked at 80 cases *per annum* in 2019. The use of femoral megaprostheses in primary hip procedures has shown a steady increase over the study period, although this increase has stagnated in recent years. Recorded use of megaprostheses in primary knee procedures increased over the study period, reaching a peak of 34 cases annually in 2017 and 2018. Nonetheless, this remained the procedure which least commonly utilizes a megaprosthesis. A steep decline in use is noted in 2020 for all femoral MPR procedure types.

**Fig. 3:**
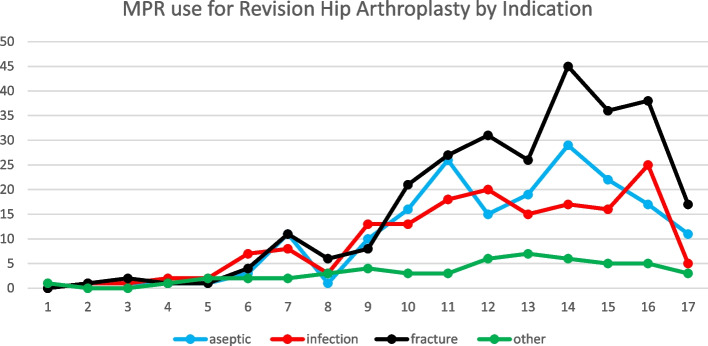
Megaprosthesis use by indication in hip revision procedures

**Fig. 4:**
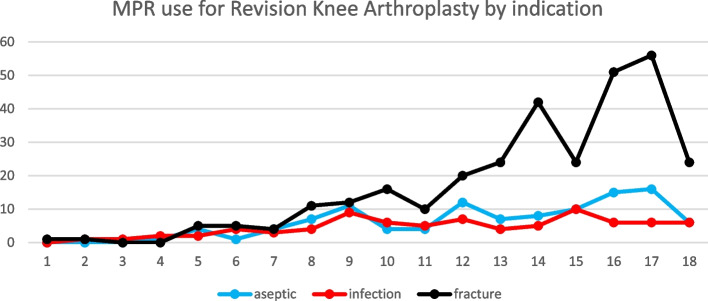
Megaprosthesis use by indication in knee revision procedures

**Fig. 5:**
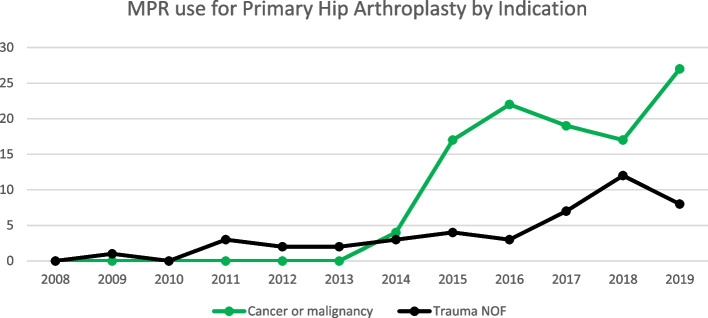
Megaprosthesis use in primary hip arthroplasty by indication

**Fig. 6:**
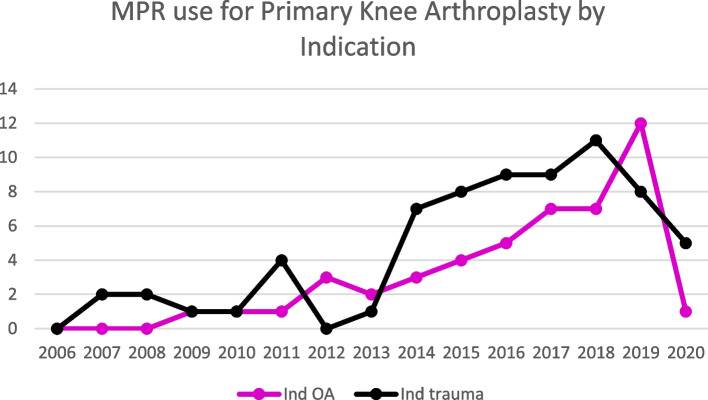
Megaprosthesis use in primary knee arthroplasty by indication

Trauma was the leading indication for MPR entries in both the hip and knee revision procedure categories, followed by aseptic loosening and infection in both groups (Figs. 3 and 4). Malignancies were the leading indication for use in primary hip arthroplasty, followed by trauma (Fig. 5). Osteoarthritis and trauma were the two leading indications for use in the primary knee arthroplasty category (Fig. 6).

MPR use for a trauma increased in all categories throughout the study period. The same is true for aseptic loosening and infection which showed a very gradual increase in incidence over the study period.

**Fig. 7:**
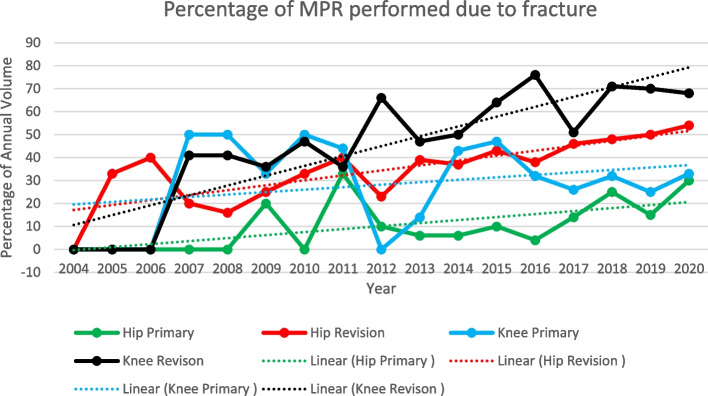
Trends in femoral megaprosthesis use for trauma indications

The proportion of cases with trauma listed as the indication was highest in the knee revision category, ranging from 68% to 75% of procedures recorded in the NJR from 2016 to 2020. The proportion of trauma cases in the hip revision category peaked at 50% in 2019. Trauma is now the leading indication submitted for megaprosthesis use in each of these categories. Femoral megaprostheses were less commonly used for trauma in the primary hip and knee procedure categories (peaking at 30% and 33% respectively) although they still showed an increase in the proportion of annual volume, in these categories over the study period (Fig. 7).

**Fig. 8:**
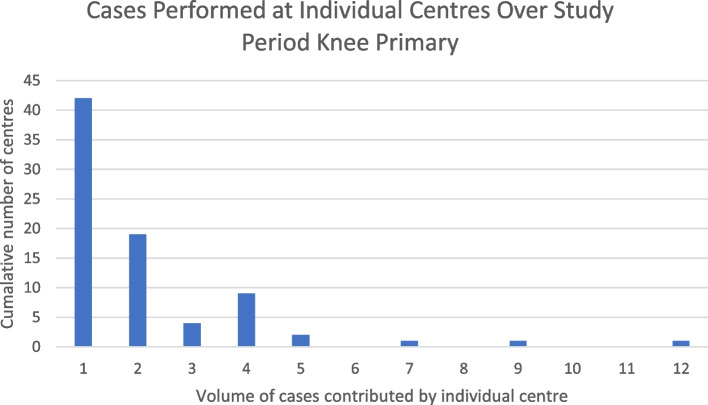
Bar chart showing the cumulative number of centers performing procedure volumes, knee primary surgery

**Fig. 9:**
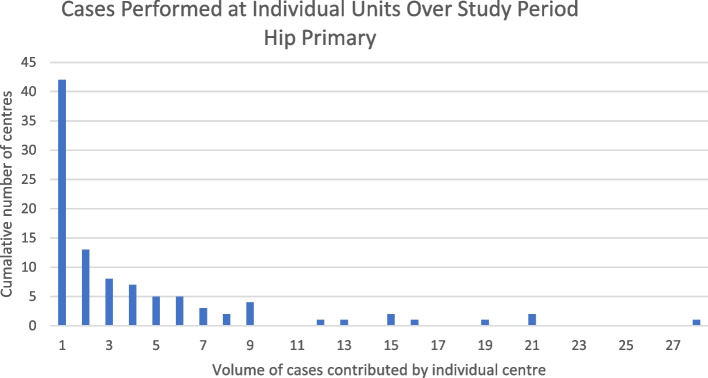
Bar chart showing cumulative number of centers performing procedure volumes, hip primary surgery

**Fig. 10:**
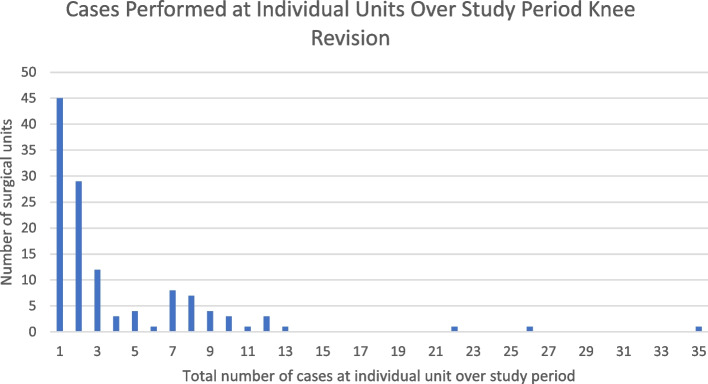
Bar chart illustrating cumulative number of centers performing procedure volumes, knee revision

**Fig. 11:**
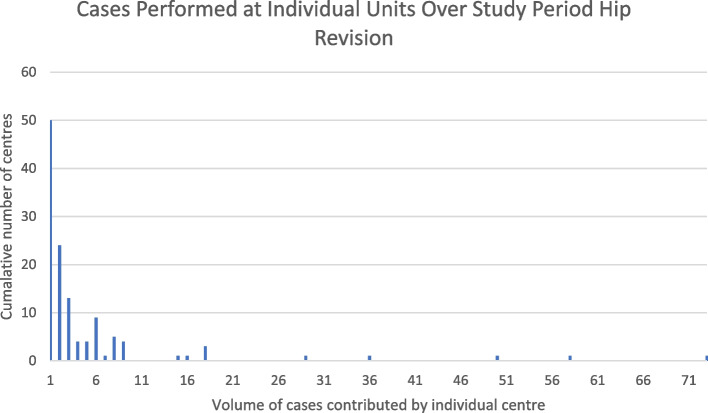
Bar chart exhibiting cumulative number of centers performing procedure volumes, hip revision

Analysis of centers at which MPRs were performed revealed low volume practice across a large number of centers. The number of individual centers which submitted data to the NJR during the study period for each procedure type is shown in Table 2. Individual centers commonly only contributed a single case in each category over the entire study period. This was the case for 42 centers in the primary hip and knee procedure categories, 45 centers in the revision knee category and 50 centers in the revision hip category. Examples of centers contributing higher volumes of cases were less common, with only 11 institutions contributing more than 20 cases to an individual NJR procedure category. The highest case volume identified consisted of 73 cases from a single centre in the revision hip category. These findings are demonstrated graphically in Figs. 8, 9, 10 and 11.

**Table 2:** Number of individual units submitting data by procedure category

Procedure Type	Number of units submitting data to the NJR
Knee Primary	79
Knee Revision	124
Hip Primary	98
Hip Revision	124

## Discussion

The primary aim of this study was to gain insight into the current usage of femoral megaprostheses in England, Wales, Northern Ireland and the Isle of Man, as recorded in the NJR. By conducting an analysis of centers currently contributing data to the NJR, we also hoped to further understand the extent to which low volume MPR practice is occurring.

Unsurprisingly, MPR use was generally highest in the revision arthroplasty categories, and in the hip revision group in particular. MPR use in the primary hip category was consistently higher than in the primary knee category throughout the study period, and, interestingly, also higher than in the revision knee category until 2017.

Excluding 2020 (when a presumed COVID-19-associated decline in megaprosthesis use was observed in all categories [11]), our data suggest that femoral megaprosthesis use, within the geographical region studied, increased between 2003–2019. Particularly, megaprosthesis use in the context of non-oncological indications rose, with trauma becoming the leading indication in both hip and knee revision categories. This finding is reflected elsewhere in the literature: currently existent center-level datasets now consist exclusively of patients undergoing femoral megaprosthesis procedures for non-oncological indications [12] and systematic reviews have also reported the outcomes of patients treated with MPR for non-oncological indications [13]. Taken together, this clearly demonstrates that the use of femoral megaprostheses has become an accepted practice in certain non-oncological scenarios. Part of the explanation for their increasing use may be the growing range of options and availability of MPRs, training exposure over time, and the increasing burden of peri-prosthetic fracture management.

It should be noted that previous single-center case series of femoral MPR use for oncological indications demonstrate higher case numbers [8, 14, 15] than whole systematic reviews investigating non-oncological uses of femoral megaprostheses [13]. This suggests that the most common indication for femoral megaprosthesis use remains malignancies. From the figures obtained by reviewing NJR data, the opposite conclusion would be drawn, highlighting the challenges faced with sporadic NJR submission.

When analyzing this dataset, the reliability of data submitted to the NJR must be considered. There are several reasons why the NJR data may not provide an accurate reflection of current trends in MPR practice. One is that surgeons may not input data for those cases likely to have a poor outcome (*i*.*e*. in cases of malignancy) due to concerns over the publication of outcomes. The likely presence of this scenario is supported by our review of published unit-level data, which suggests that several large tertiary referral centers are performing many more MPRs than recorded in the NJR. One example is a retrospective review of instability following megaprosthesis use for proximal femoral tumors comprising of 527 patients treated at a single institute [11]. Considering this, we recognize that the true accuracy of the data and trends presented here are difficult to ascertain in the context of non-mandatory submission. Complicating MPR submission to the NJR further are several issues relating to how megaprosthetic procedures fit with the existing NJR data entry structure. For example, a total femoral replacement would require submission of both hip and knee forms, hemiarthroplasty articulations are not technically covered by the current system, and diaphyseal procedures have no mechanism for registration. These limitations can be addressed and overcome by the use of a megaprosthesis-specific data entry form.

Another important finding is the apparent large number of centers identified which have performed very low numbers of femoral MPRs. In all procedure categories, vast numbers of centers submitted just one case to the NJR, over the 17-year period. A similar finding was observed when this analysis was repeated for data from 2015–2020 only, an analysis we chose to perform to discard the NJR's early years when submission rates were lower than they are currently. These findings strongly suggest that current practice is at odds with the recommendations made as part of the Getting It Right First Time (GIRFT) review, which recommends that activities of high complexity should be concentrated in specialist units, within regional networks, to provide the best possible patient care [16, 17]. The volume of units that have submitted data in recent years suggest that further action is needed to ensure that GIRFT recommendations are met for megaprosthesis procedures. These findings are similar to those identified by the working group in revision knee surgery, when analyzing the provision of revision knee surgery in England, Wales, Northern Ireland and the Isle of Man [18], highlighting the fact that this is unlikely to be an isolated issue relating to MPR procedures. Non-specialist orthopedic services should seek to reduce the level of low volume practice highlighted in this study, and this may be achieved, in part, by the ongoing evolution of the regional networks and centralization of specialist care provision.

Although the true volume, indications and breadth of care provision for MPR work is difficult to ascertain from review of NJR data, findings identified within this study remain relevant to orthopedic practice and to help drive quality improvement. It is clear from the data presented that femoral MPRs were used for a wide variety of indications, beyond the oncological reconstructions for which they had been originally designed. The under-reporting highlighted in this study and the described reasons for this demonstrate the need for changes in the way that MPR procedures are entered into the registry to facilitate the monitoring of implants, meaningful research and the monitoring of specialist service provision. To facilitate this, a megaprosthesis-specific NJR data entry form has been developed for implementation into clinical practice.

Additionally, support is given for amendments to the processing of all procedures with malignancies as the given indication, so that these cases do not contribute to individual, unit or global outcomes. This amendment would provide surgeons with the confidence to accurately report all such procedures being performed, and in doing so create opportunities for meaningful research and improve the understanding of specialist care provision nationally.

## Conclusions

This analysis demonstrated that submissions of femoral megaprosthesis cases to the NJR are increasing, particularly for non-neoplastic indications. The data also suggest that a large number of centers have been performing low volumes of femoral MPRs, which is not in keeping with GIRFT recommendations. However, clear disparities between NJR data and other published literature suggest a significant degree of under-reporting of megaprosthesis cases to the NJR, therefore, these results must be interpreted with caution. To address this, we advocate the use of MPR-specific registry data entry forms, and for special consideration to be given to how data are analyzed and reported for cases where malignancy is stated as the indication for surgery.

## Supplementary Information

**Additional file 1.**

## Data Availability

Available upon request.
