# Supplementary figures and images for: Apparent trends in the use of femoral megaprostheses: an analysis from the National Joint Registry
Source: Arthroplasty. 2022 Dec 1;4:50. doi: 10.1186/s42836-022-00150-7 (PMC9713154; doi:10.1186/s42836-022-00150-7)

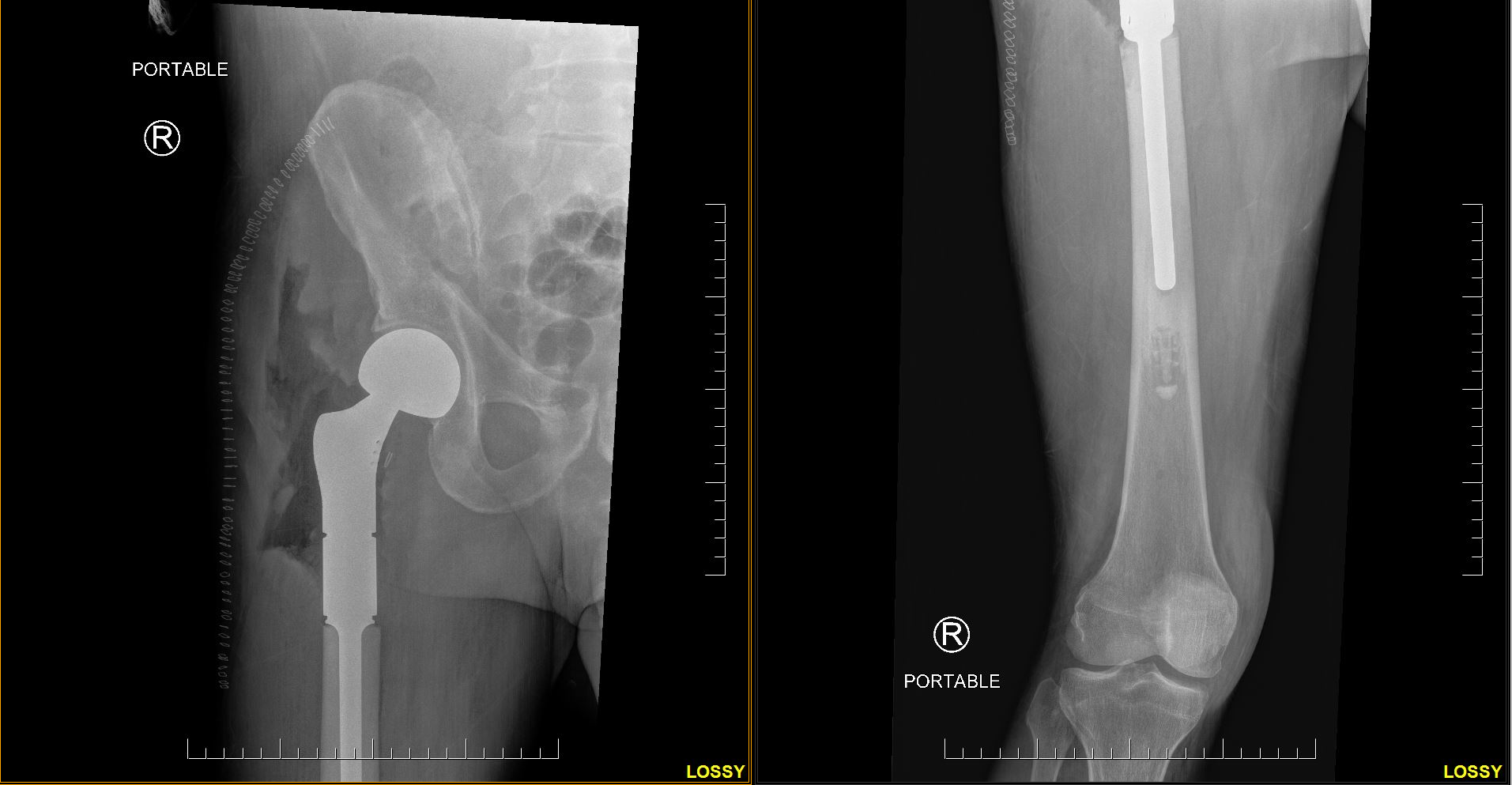

Supplement: Supplementary file 1 — Additional file 1. [file 42836_2022_150_MOESM1_ESM.png]
